# Management lessons through an interactive online discussion about hospital management during the COVID-19 pandemic

**DOI:** 10.3205/zma001421

**Published:** 2021-01-28

**Authors:** Alexander Leunig, Markus Winkler, Jonathan A. Gernert, Tanja Graupe, Konstantinos Dimitriadis

**Affiliations:** 1 University Hospital of Ludwig-Maximilian-University, Institute for Medical Education, Munich, Germany; 2 University Hospital of Ludwig-Maximilian-University, Neurologische Klinik und Poliklinik, Munich, Germany; 3 University Hospital of Ludwig-Maximilian-University, Institute for Stroke and Dementia Research (ISD), Munich, Germany

**Keywords:** medical education, COVID-19, hospital management, digital education, healthcare policy, podium discussion

## Abstract

**Background:** In light of the COVID-19 pandemic and resulting demand for innovative hospital management we organized an interactive online discussion for medical students and healthcare professionals about hospital management during the crisis.

**Objective:** The event offered an opportunity to learn from a hospital crisis management. We looked at how this new online format compares to a traditional discussion event.

**Methods:** We used an online platform with four guests, a moderator and about 100 attendees. During the event we gathered demographic facts through an interactive questionnaire tool and an extensive evaluation afterwards.

**Results: **The event was rated with an overall grade of 1.4 (Likert from 1 to 6, 1 best grade; SD 0.5) and participants agreed that this format should be organized again (1.2; SD 0.5). 70% of audience members preferred the online format of the event. Due to the high volume, only about 30% (total n~35) of the questions posed by the audience were addressed.

**Conclusion: **Firstly, most participants preferred the event to be online, contrary to our expectation. Secondly, the handling of the amount of individual questions posed significant challenges.

Finally, the number of attendees and questions suggested a continuing demand among students and physicians for further education regarding hospital management, especially regarding COVID-19.

These findings also require a critical look at future formats and topics of podium discussions in medical education. The online format might be a good alternative to face-to-face lectures.

## Zusammenfassung

**Hintergrund: **Die COVID-19-Pandemie und die daraus resultierende Nachfrage nach innovativem Krankenhausmanagement hat uns dazu bewogen, eine interaktive Online-Diskussion für Medizinstudierende und Angehörige der Gesundheitsberufe zum Thema Krankenhausmanagement während der Krise zu organisieren.

**Zielsetzung: **Das Ziel der Veranstaltung war aus dem Krankenhauskrisenmanagement zu lernen. Dazu haben wir unser neues Online-Format mit einer traditionellen Veranstaltung verglichen.

**Methoden: **Wir haben eine Online-Plattform mit vier Gästen, einem Moderator und etwa 100 Teilnehmern aufgesetzt. Während der Veranstaltung haben wir mit Hilfe eines interaktiven Fragebogen-Tools und einer anschließenden umfangreichen Teilnehmerbefragung demographische Daten gesammelt.

**Ergebnisse:** Die Veranstaltung wurde mit einer Gesamtnote von 1,4 bewertet (Likert von 1 bis 6, 1 beste Note; SD 0,5) und die Teilnehmer haben sich dafür ausgesprochen, dass dieses Format erneut organisiert werden sollte (1,2; SD 0,5). 70% der Zuhörer bevorzugten das Online-Format der Veranstaltung. Aufgrund der hohen Zahl an gestellten Fragen konnten nur etwa 30% (insgesamt n~35) der vom Publikum gestellten Fragen angesprochen werden.

**Fazit:** Erstens bevorzugten die meisten Teilnehmer, entgegen unserer Erwartung, die Online-Veranstaltung. Zweitens stellte die Bewältigung der hohen Anzahl an Fragen eine große Herausforderung dar. Drittens deuten die hohe Anzahl an Teilnehmern und Fragen auf eine anhaltend hohe Nachfrage von Studierenden und Ärzten nach Weiterbildung im Bereich des Krankenhausmanagements, insbesondere in Bezug auf COVID-19, hin.

Diese Ergebnisse erfordern auch einen kritischen Blick auf zukünftige Formate und Themen von Podiumsdiskussionen in der medizinischen Ausbildung. Das Online-Format könnte eine gute Alternative zu Präsenz-Vorträgen sein.

## Introduction

MeCuM-SiGma (Medical Curriculum Munich – Simulation Healthcare Management) is a course designed to teach the basics of healthcare management and policy to medical students [1], [2], [3]. The course consists of weekly problem-based seminars, lectures and a simulation where students play the role of a hospital advisory board and work on a project selected by the board of directors. The course also hosts a yearly summer event on current healthcare policy and management topics. MeCuM-SiGma is well evaluated and provides experience for medical students in healthcare management [4], [5].

## Project description

The COVID-19 pandemic made increasingly clear that hospital management is vital in preventing shortages, allocating staff efficiently and providing the best care possible in an overextended healthcare system [6], [7]. It offered an opportunity to draw lessons from the experience and use it as a case study to pass on insights into hospital management issues. Hence, we organized an online event called: “Crisis management at university hospitals - looking back and lessons for the future”. We chose a format of a virtual moderated podium discussion with questions from the audience. An in-person format was evaluated positively in the past and seemed suitable for our learning objectives [8], [9], [10]. We sought to use this successful in-person format for an online event. The event had to include a podium discussion, a moderator and interactive audience participation to be comparable. The CEO and chief physician of a university hospital, the chief nursing officer, the head of ICU-response, and a representative of the health department were guests. A current medical resident, who was involved in the COVID-19 response, moderated the discussion. The event began with 5-minute introductions from each guest, where they detailed their management experience during the crisis. The rest of the discussion was driven by questions from the audience.

The entire event was held via an online platform (Zoom). The audience was muted for the duration of the event and only submitted questions via chat. The total length of the event was about 2 hours. During the event, we gathered demographic facts about the audience using an interactive questionnaire (60% response rate). Participants also completed a post-event evaluation form (30% response rate).

## Results

142 people registered for the event. The maximum number of simultaneously logged in guests was 100. At 1,5 hours some participants left the chatroom, 70 participants remained until the end. 80% of the respondents of our live survey were medical students and 16% were physicians. Most participants did not have any hospital management experience (69%). In the follow-up evaluation (n=30) the mean age was 30 years (SD 10) and 47% of participants were female. The event was rated with an overall grade of 1.4 (Likert from 1 to 6, 1 best grade; SD 0.5) and participants agreed that this format should be organized again next year (1.2; SD 0.5). The grading was comparable to four similar in-person events (1.52±0.7, 1.41±0.71, 1.38±0.49 and 1.6±0.7). 69% of participants stated that they were doing something else on their computer and they generally rated the possibility of parallel work as positive (2.5; SD 1.5). Surprisingly, 70% of audience members preferred the online format. Participants indicated that they were unhappy with the handling of the posed questions. 35 questions were asked, 40% about hospital management during the crisis, 20% about future planning, 20% about policy and 20% other categories. Few questions were asked about personal or student topics. Due to the high volume of questions the moderator could only address about 30% of the questions posed.

## Discussion & conclusion

This case report is about a one-time event; hence we are cautious to draw general conclusions. However, our results show some interesting trends. The event was evaluated positively. Participants favored being able to complete other tasks simultaneously. It has been shown before that online multitasking is more common than at in-person events [11], also that online learning for medical students is noninferior to in-person learning [12]. This might be the reason why participants preferred an online event, contrary to our expectation and institutional norm. Some selection bias is likely, as only students that participated in this online discussion were asked. Future surveys about the preferred mode of discussion rounds for all students are needed. Clear communication of a time concept seems important, as more participants logged out towards the end of the event.

The handling of questions posed significant challenges. Contrary to classroom events, where questions are asked individually, new online formats, especially chat boxes, allow a simultaneous posing of questions. Although many audience members asked questions (ca. 35%), indicating high engagement, the participants were unhappy that many questions went unaddressed. Based on our experience organizing similar in-person events, less questions are asked in person even when the overall ratings of the event are comparable.

The number of attendees and questions asked suggests a robust demand among medical students and physicians for further education regarding hospital management, especially on the topic of COVID-19. These findings require a critical look at the format of future podium discussions in medical education. Many participants preferred the online format and topic. Online discussions can be a good alternative to, or additional feature of, in-person lectures even post-COVID-19. It is advisable to have a concept to deal with a high volume of questions during online discussions. In the future we will collect unanswered questions and ask guests to answer them in written form or as a podcast after the event. In regard to learners’ attention and amount of questions, it remains to be examined whether the anonymity of an online event is advantageous.

## Competing interests

The authors declare that they have no competing interests. 
